# Addressing Potential Cumulative Impacts of Development on Threatened Species: The Case of the Endangered Black-Throated Finch

**DOI:** 10.1371/journal.pone.0148485

**Published:** 2016-03-02

**Authors:** Eric Peter Vanderduys, April E. Reside, Anthony Grice, Juliana Rechetelo

**Affiliations:** 1 CSIRO Land and Water, PMB PO, Aitkenvale, Queensland, Australia; 2 Centre for Tropical Environmental & Sustainability Sciences, James Cook University, Queensland, Australia; 3 College of Marine and Environmental Sciences, James Cook University, Queensland, Australia; Université de Sherbrooke, CANADA

## Abstract

Where threatened biodiversity is adversely affected by development, policies often state that "no net loss" should be the goal and biodiversity offsetting is one mechanism available to achieve this. However, developments are often approved on an *ad hoc* basis and cumulative impacts are not sufficiently examined. We demonstrate the potential for serious threat to an endangered subspecies when multiple developments are planned. We modelled the distribution of the black-throated finch (*Poephila cincta cincta*) using bioclimatic data and Queensland's Regional Ecosystem classification. We overlaid granted, extant extractive and exploratory mining tenures within the known and modelled ranges of black-throated finches to examine the level of incipient threat to this subspecies in central Queensland, Australia. Our models indicate that more than half of the remaining *P*. *cincta cincta* habitat is currently under extractive or exploratory tenure. Therefore, insufficient habitat exists to offset all potential development so "no net loss" is not possible. This has implications for future conservation of this and similarly distributed species and for resource development planning, especially the use of legislated offsets for biodiversity protection.

## Introduction

The high rates of biodiversity decline documented globally demand close attention to the conservation status of threatened species where threats are ongoing [1, 2]. Many species continue to decline because of changes in land use that include broad scale land clearing for agriculture and urban development, as well as more subtle effects from fragmentation, invasive species, grazing, changed fire regimes and shifting climate envelopes [3].

Globally, offsets are seen as a mechanism for achieving no overall net loss or net environmental gains in the face of development pressures (see summaries and examples in [4–8]). In Australia, where threatened species and ecosystems (e.g. matters of national environmental significance (MNES); [9, 10]) are likely to be adversely affected by development, state and federal legislation (e.g., [9, 11, 12]) dictates that development proponents must show that impacts will be offset. This may mean compensating for MNES losses to achieve no net loss [9, 13] by implementing management to maintain or improve viability and provide equivalence for the MNES [11, 14]. However, it is difficult to compensate for loss of threatened species or their habitat; particularly for species with very specific requirements, species' whose requirements are poorly known, or where suitable habitat cannot readily be recreated [15]. Thus, avoiding loss of the habitat of threatened species to achieve the goals of the acts, legislation and policies listed above is logistically problematic [6], and evaluating whether this loss has been avoided is equally fraught [16–18]. Creating offsets that reliably avoid loss of threatened species habitat, particularly when known areas of high habitat suitability are compromised, lacks both theoretical support and practical methodology for the majority of species.

It is well recognised that potential cumulative impacts [19] are often overlooked when multiple developments are considered in isolation and the landscape context is ignored [20], decreasing the likelihood of achieving a "no net loss" outcome. Defining appropriate reference frames and counterfactual scenarios is critical to effective offsetting [21, 22] as are timeframes for establishing [6] and maintaining the benefits of the offsets [22]. A precautionary approach is critical because "when offsetting is proposed, impacts to biodiversity are certain and effective offsets are not" [23]. Both statutory (strategic assessments; [24]) and non-statutory (e.g. Galilee Basin Offset Strategy; [12]) instruments exist to help plan within a landscape context. Four strategic assessments are in place and twelve in progress across Australia [24, 25]. However, there is no requirement to coordinate offset selection, tenure arrangements and maintenance of offsets outside of strategic assessment frameworks.

We examine the extent of potential cumulative impacts of mining industry activities in the case study of a threatened bird, the black-throated finch *Poephila cincta cincta* (hereafter BTF). To date, there has been no investigation into whether there would be sufficient habitat left and available to be used as offset, should all the planned mining activities proceed. Without this investigation, there can be no real understanding of the probability of persistence of BTF. This is symptomatic of development procedure in Australia and elsewhere, so we aim to use the case of the BTF to highlight the importance of investigating cumulative impacts on threatened species more generally.

BTF inhabit open woodlands and forests with seeding grasses and free water (summarised in [26]) and generally take their food, primarily grass seeds, from the ground [27]. Most aspects of BTF social structure and breeding behaviour in the wild are poorly known [26]. Nests are usually in trees, shrubs, mistletoes, raptor nests or tree hollows [26, 27] and clutch size varies from three to nine [26]. Patterns of movement across the landscape, if they occur, are poorly known [28] although two sightings of one banded bird were 16 km apart [29]. There is no evidence for larger scale movements across the BTF's range.

The extent of occurrence of BTF has contracted by an estimated 80% over the last 30 years [26, 30]. Declines have been linked to land use change, primarily land clearing and intensification of livestock grazing [26, 30–32], conversion of habitat to pasture, including the introduction of non-native fodder grasses, fragmentation, weed invasion, urban expansion and synergistic effects involving these factors, drought and fire [30–32]. These forces have occurred in an area that has undergone "one of the most rapid landscape transformations ever documented" (see [33] and references therein). Within BTF range in Queensland, vegetation clearing has been relatively more extensive in the south than the north [34] and past clearing has been coincident with BTF declines (compare [30, 33, 35, 36]), especially in the Brigalow Belt bioregion where over 60% of the original vegetation has been cleared [33, 37]. BTF are not known to have declined within the northeast Desert Uplands, an area subject to relatively less land clearing than other parts of BTF range [34]. There are extensive resource extraction leases and exploration permits in this region [38], representing a hitherto unrealised threat to BTF. Planned and approved developments include large open-cut mines (e.g. 24 km long [39]), underground mines subject to post-mine subsidence [40, 41] and associated infrastructure.

Because of its endangered status, offsets are being considered in the face of resource extraction developments planned in remaining BTF habitat (e.g., [12, 42]). However, each development is considered individually, and there has been no attempt to estimate the potential impacts on BTF of all the developments in concert. We examine the total footprint of all the resource extraction and exploration leases in BTF habitat and from this we identify the amount of BTF habitat available that could be used for offsetting purposes. We argue that there is insufficient land that is BTF habitat available for offsetting, even though this approach to offsetting has been criticised as being logically flawed [43]. We also argue that there is insufficient land available for an offsetting scheme where restoration or habitat improvement is the mechanism used to create the offsets if all the planned developments go ahead.

## Methods

### Species data

**Fig 1:**
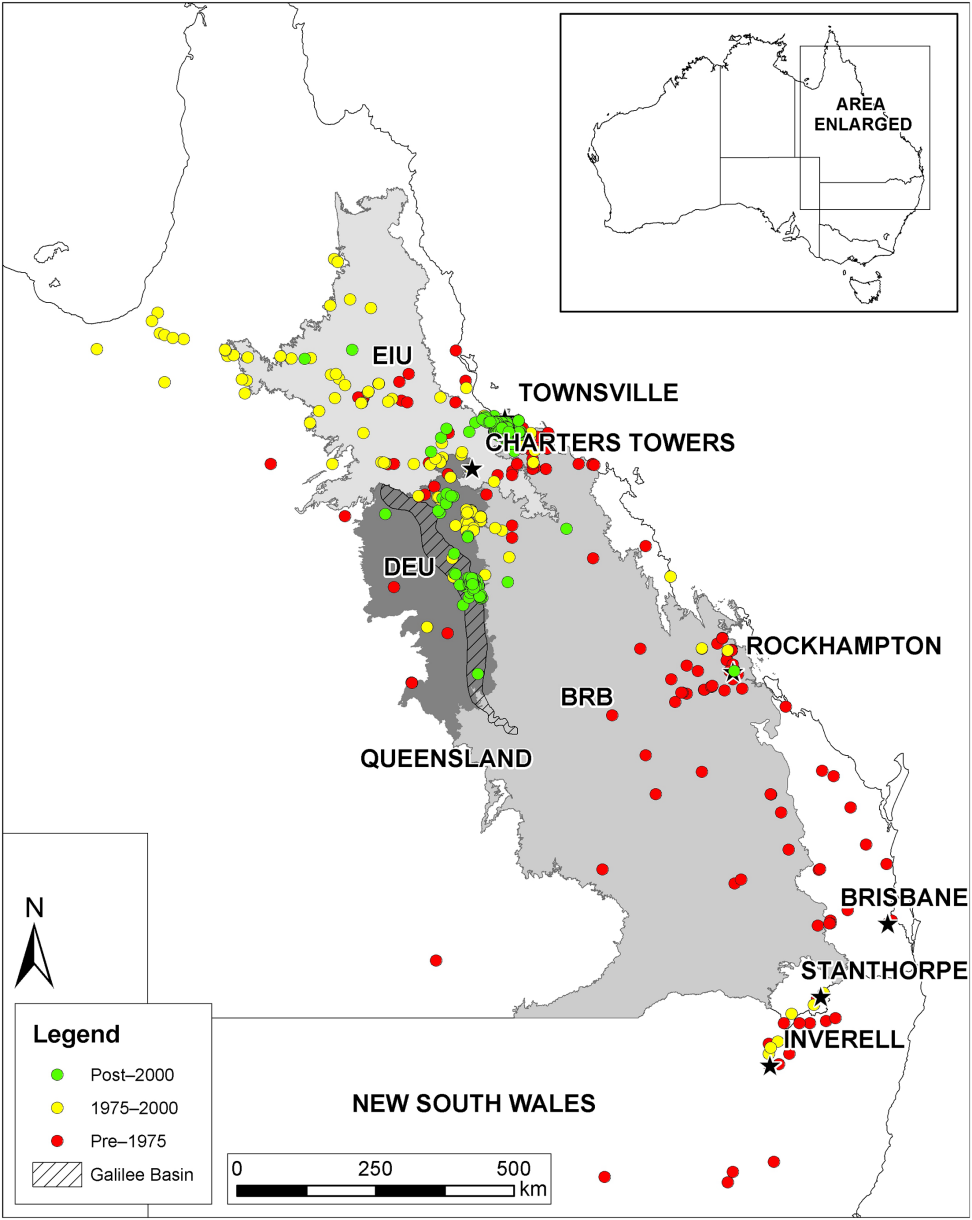
Distribution of BTF records colour-coded by years. BTF records from all year-classes were used to create the bioclimatic model. The most relevant IBRA bioregions are shaded: BRB = Brigalow Belt; DEU = Desert Uplands; EIU = Einasleigh Uplands. Relevant towns and the approximate extent of the Galilee Basin coal measure (from [77]) are shown. Used under a CC BY license, with permission from Eric Vanderduys, original copyright 2015.

The black-throated finch is an Australian estrildid grass-finch consisting of two subspecies, the northern: *Poephila cincta atropygialis*, confined to Cape York Peninsula and the northern and western Gulf Plains, QLD; and the southern subspecies: *Poephila cincta cincta*, now largely restricted to the Townsville Plain and portions of the Desert Uplands and Brigalow Belt bioregions, QLD, south to about 23°S (Fig 1). The northern subspecies is not listed as threatened, while the southern subspecies is listed as endangered under the Federal *Environment Protection and Biodiversity Conservation Act* [10], the *QLD Nature Conservation Act* [28] and the New South Wales *Threatened Species Conservation Act* [44]. BTF once occurred as far south as 31°S in New South Wales (NSW), but there are no recent records and it may now be extinct in NSW [26]. In the vast area of its former range in southern QLD (south of 23°S) there have been only nine records since 1980 ([45], QLD Wildnet and Black-throated Finch Recovery Team databases, unpublished data), including one in 1990 from near Stanthorpe in the extreme south and one at Rockhampton in 2004 (Fig 1). BTF now appear to have two major strongholds—parts of the Townsville Plain in the northern Brigalow Belt bioregion, and along the eastern edge of the Desert Uplands bioregion (see [46]).

BTF records were accessed from Birdlife Australia [45, 47], CSIRO Ecosystem Sciences [48], the Black-throated Finch Recovery Team (BTFRT, unpublished data, 2014) and ongoing research [29], environmental impact statement data available on the internet [49–51], and data acquired through a 10-year water source monitoring program co-ordinated by the BTFRT on the Townsville Plain. Data were vetted by eliminating northern subspecies records, plus any listed as unspecified subspecies north of 18.9° S, which is the approximate northern limit of the southern subspecies. Three BTF records that fell in the Pacific Ocean were assumed to be errors and removed. Five records of unspecified subspecies BTF from 1880–1928 in the western edge of the species' range (north west Queensland) were removed because they were surrounded by more contemporary northern subspecies records and literature suggests only the northern subspecies occurs that far west [27]. In order to account for spatial bias in the occurrence data [52] because of unequal sampling effort (largely a result of proximity to an urban centre), we employed the following two spatial thinning methods: the vetted BTF records were rounded to both two and three decimal places, and any duplicate locations removed after these rounding processes. These roundings amount to roughly 700 m and 70 m, respectively, across BTF range. Rounding left us with totals of 466 and 850 BTF record locations for two and three decimal places, respectively. While rounded duplicates were removed, the original precision was retained in the remaining occurrence data for running the models. The BTF records shown in Fig 1 are the three decimal place rounded records. The two decimal place records appear the same at the scale shown in Fig 1.

### Climate data

Climate data were derived from ANUCLIM [53] at a 9-second resolution (approximately 250 m grids). The climate variables used were 30-year averages for the period 1976–2005 of annual mean temperature, temperature seasonality, maximum temperature of the warmest period, minimum temperature of the coldest period, annual precipitation, precipitation of the wettest period, precipitation of the driest period and precipitation seasonality. These variables have been shown to represent the bioclimatic limits of vertebrates throughout this region [54–62].

### Modelling protocol

The BTF climate envelope was modelled using Maxent [63]. In addition to rounding occurrence data described above, to further account for spatial bias in the occurrence records, we used target-group background points in Maxent [64]. Background points were derived from records of common mynas (*Acridotheres tristis*) and six sympatric woodland species: weebills (*Smicrornis brevirostris*), squatter pigeons (*Geophaps scripta*), peaceful doves (*Geopelia striata*), double-barred finches (*Taeniopygia bichenovii*), black-faced woodswallows (*Artamus cinereus*) and little woodswallows (*A*. *minor*), obtained from the online Atlas of Living Australia [65], systematic data collection sites [48], standardised water source counts and camera trap observation points [49, 66]. The species in the the target-group background were chosen because they: are strongly associated with humans (common myna [26]), and thus likely to account for spatial bias of species records around urban centres and along roads [67]; are widespread and common over much of Australia (weebill, peaceful dove [68, 69]); share a similar but not so dramatic pattern of decline to BTF (squatter pigeon [69]); are granivores, often occurring in similar areas and habitat to BTF (squatter pigeon, peaceful dove, double-barred finch [26, 68, 69]); are known to associate with BTF in mixed flocks (weebill, woodswallows, double-barred finch [26, 70]); are of a similar size to or smaller than BTF (double-barred finch, weebill [26, 68]) and often cryptic (weebill). By using the species above, and clipping target-group background points to QLD and NSW, we addressed the tension between overly generous environmental space represented in the background points and environmental space too similar to that of BTF occurrences [60]. Background points were rounded were rounded and treated as the BTF points for the model runs. Maxent used 99100 and 99334 points (background plus BTF presence points) to create the two decimal and three decimal place distribution models, respectively.

We ran the model twice: first with the two decimal place occurrence records, and second with the three decimal place occurrence records. This allowed us to investigate the trade off between levels of spatial thinning and sample size. Initially, the suitable climate space for BTF was designated as the area within the two and three decimal place Maxent-derived climate envelopes that had suitability scores ≥0.5. Both models were clipped, so that below the threshold suitability became zero, and above the threshold was assumed to be climatically suitable. We validated the models by area under the receiver operating characteristic curve (AUC) [71].

Because much of BTF range is within vast tropical and subtropical savannas and open woodlands, which generally lack dramatic topography and have diffuse bioclimatic boundaries, we refined the models by clipping the climatically derived distribution models as follows. We first removed from the climate envelope islands and built up areas of cities and towns where no BTF have been recorded. We then brought in fine-scale vegetation mapping and land-use data in order to achieve a realised species distribution approaching BTF's area of occupancy. To do this, we determined the primary Regional Ecosystem (RE, as defined in "description" in the Regional Ecosystem Description Database [72, 73]) designation underlying each BTF record within the suitable climate modelled areas. This step in the modelling process is important because fine-scale species-habitat associations are necessary in deriving actual area of occupancy of a species [74] and BTF are known to use clumped resources or use resources in a clumped fashion [75]. We added in the RE classification in the post-model processing because RE classifications are mapped at a much finer scale than the 250 m grid squares used in the bioclimatic modelling process and to upscale REs to 250 m would have resulted in loss of fine-scale resolution. Primary REs with three or fewer (unrounded) presence records were ignored as being unlikely to be favourable habitat and areas listed as water or cleared (non-remnant) were also ignored. There were 23 REs within the area that had a bioclimatic suitability of ≥0.5 for the two decimal place model and 21 REs within the area of ≥0.5 suitability for the three decimal place model. Recognising that black-throated finches may sometimes use cleared areas in proximity to suitable habitat (i.e. suitable REs), we buffered suitable REs to a distance of 1118 m. This distance was chosen as an average of the maximum distances travelled by 15 radio-tracked BTF on the Townsville Plain [29]. To address the tension between omission and commission errors [74], omission error rates were calculated by checking each of the models against recent records, which were defined as being from the year 2000 or later (2312 records). Although Rondinini et al. [74] state that it is impossible to quantify omission and commission error rates for opportunistically collected records (as many of the BTF records were), examining the spatial locations of BTF records and background points relative to the two models is useful, as it suggests where errors might exist. Commission error rates were estimated by checking each of the models against the 99064 background points. Using these processes we chose a final habitat model based on bioclimatic and specific mapped Regional Ecosystems, buffered to 1118 m referred to as the "habitat model". Because the Regional Ecosystem classification does not indicate pre-clearing vegetation type, we used broad vegetation group classification (BVG [76]), overlaid with non-remnants in the Regional Ecosystem classification, to determine potential cleared extents available for rehabilitation as offsets. We recognise that BVG descriptions lack the detail of RE descriptions, but this was the most systematic way to assess potential offset areas.

### Resource extractive and exploratory industries extents

The BTF range overlaps with the Galilee Basin coal measure (the 'Galilee Basin'), an approximately 500 km long thermal coal deposit running roughly north-south, in central QLD (Fig 1; [77]). However, we discuss resource extraction and exploration more generally in this paper.

In QLD there are a number of extractive (mining) permit and lease types, governed by the *Mineral Resources Act 1989* and the *Petroleum and Gas (Production and Safety) Act 2004* [78, 79]. These range from permits that allow exploration activities to occur in a given area, up to permits for resource extraction. Exploration permits are no guarantee of future realised resource extraction, but do require that on-ground activities such as a drilling program are conducted in a timely fashion as detailed in a work program to be submitted to the Department of Natural Resources and Mines [80]. From Queensland Spatial Catalogue Data [38], we accumulated granted extractive and exploratory industry tenures (Exploration Permits for Coal (EPC), Exploration Permits for Geothermal (EPG), Exploration Permits for Mineral (EPM), Exploration Permits for Petroleum (EPP), Mining Claim (MC), Mineral Development Licence (MDL), Mining Lease (ML), Petroleum Lease (PL), Petroleum Survey Licence (PSL)) for QLD that were extant as of 7 September 2015. We calculated the proportion converted from one permit or lease type to another because areas subject to exploration permits have a lower chance of resource realisation than areas covered by extraction leases (e.g. MLs). We examined proportions of each tenure type in relation to modelled BTF habitat in order to gain a realistic understanding of potential threats to BTF. In parts of the southern and central Galilee Basin, some MLs and MDLs have been granted over exploration permits and proponents have submitted detailed plans of proposed impact areas [38–41, 81–83]. To calculate the conversion rates from lower likelihood (e.g. EPC) to higher likelihood (e.g. ML) tenure, we included some expired exploration leases (i.e. expired before 7 September 2015; [38]) because these underpin the subsequent detailed plans referred to above. Impacts resulting from these developments are likely to include broad scale clearing and conversion to large open-cut coal mines in a roughly north-south orientation, with extensive areas of underground mining to the west of this. Plans also include clearing for infrastructure such as airports, railways and accommodation areas and underground mined areas are likely to be subject to subsidence (e.g., [40, 41, 81]). We mapped the planned extents of these developments using geo-rectified images in ArcGIS 10.2 and compared these extents to tenure maps available from [38]. We overlaid the BTF habitat model with this resource tenure information to determine the overlap of these potential land uses with BTF habitat. We also overlaid the habitat model on the protected area (National Park) estate within QLD [84], to determine its current protected extent.

We used these same resource tenure data in relation to the Galilee Basin Offset Strategy (GBOS [12]). The GBOS identifies three priorities that make up a strategic footprint within the northern Brigalow Belt and Desert Uplands bioregions: 1) high conservation value areas; 2) key north-south and east-west corridors that link to adjacent bioregions; and 3) areas with potential for rehabilitation, that is, for offsetting. We refer to these subsequently as Priority 1, 2 and 3 areas.

## Results

The Maxent climate envelope models had high performances with AUCs of 0.95 and 0.97 for the two and three decimal place models, respectively. Both of the models had very low omission rates with 99.0% (2288/2312) of the recent records falling within the two decimal place model, threshold (i.e. climate suitability ≥0.5), while 94.7% (2189/2312) of the recent records fell within the three decimal place model.

When unsuitable areas such as islands and built up urban centre were removed, and the suitable RE classifications with a 1118 m buffer were added to the climate models, model omission rates were still very low, with 98.4% (2276/2312) of the recent records falling within the two decimal place model threshold (i.e. climate suitability ≥0.5), and 96.6% (2233/2312) of the recent records falling within the three decimal place model.

**Fig 2:**
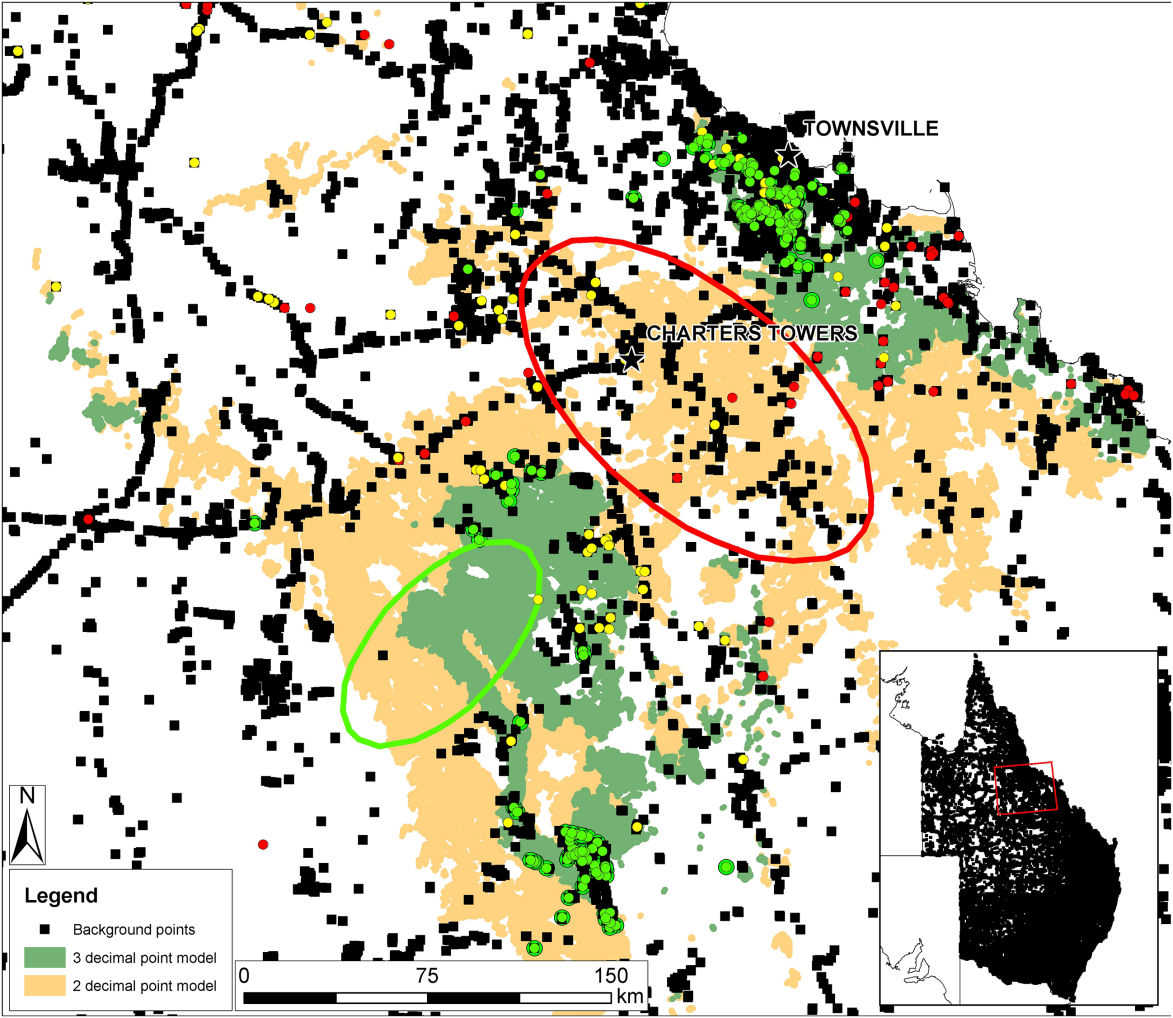
Map showing areas of potential commission and omission errors in the two decimal place and three decimal place models. The red ellipse enclosing the city of Charters Towers shows a large area of potential commission errors in the two decimal point model, with 282 target-group background points (i.e. bird records used for the background), with 14 BTF records, all pre-2000. The green ellipse shows a very poorly sampled area. Green, yellow and red dots are BTF records using the same schema as in Fig 1. Inset map shows the 99,000+ target-group background points used in constructing the models. Used under a CC BY license, with permission from Eric Vanderduys, original copyright 2015.

Although absences for mobile organisms are particularly difficult to ascertain [85], more than double the number of background points fell within the two decimal point plus RE model (1919) than the three decimal point plus RE model (976 background points) (Fig 2). Importantly, large areas of the two decimal point model had many background points and no BTF records, suggesting potential commission errors in this region. Furthermore, the total area of the two decimal point model was 50,025 km^2^, while for the three decimal point model it was 15,563 km^2^. For a gain in area of over three times, the two decimal point model loses 43 post-2000 omission records of BTF, an improvement of 1.9% over the three decimal point model. Thus, because the three decimal place model has no obvious areas with many background points suggesting commission error, and because of the vast geographical area required to remove a small percentage of omission errors, we have adopted the three decimal place model as the most realistic model of the area of occupancy for BTF. This is the "habitat model" hereafter.

Total areas of the non-buffered REs were 6,821 km^2^. A breakdown of the component favourable RE extents is given in Table 1, along with the numbers of post-2000 BTF records within each RE. Two Townsville Plains REs (11.3.30: *Eucalyptus crebra*, *Corymbia dallachiana* woodland on alluvial plains and RE11.3.35: *E*. *platyphylla*, *C*. *clarksoniana* woodland on alluvial plains [73]) contained 74.5% of the post-2000 BTF records. In the less sampled western part of BTF range the dominant two BTF REs (10.5.5a: a variant of *E*. *melanophloia* open woodland on sand plains and 10.3.28a: a variant of *E*. *melanophloia* or *E*. *crebra* open woodland on sandy alluvial fans [73]) accounted for 4.2% of the post-2000 records.

**Table 1:** Areas of modelled favourable Regional Ecosystems (RE; see Methods, *Modelling protocol*) within the three decimal point bioclimatic model, areas currently mapped as remnant, and areas not covered by extant mining or exploration leases. The number of post-2000 BTF records for each RE are given and IBRA subregions [46] are shown. Some REs have no post-2000 BTF records but are included as favourable BTF REs because there were >3 older BTF records from those REs. The habitat model referred to is the 1118 m buffered REs.

RE	Total available (km^2^)	Area with no mining interest (km^2^)	% no mining interest	Number of post-2000 BTF records	IBRA subregion
7.12.24a	4	4	100.0	0	Townsville Plains
7.12.65b	1	1	100.0	0	Townsville Plains
9.8.1a	113	10	9.3	0	Cape-Campaspe Plains, Undara—Toomba Basalts
9.12.1a	4	4	100.0	0	Broken River
10.3.6a	840	101	12.0	1	Cape-Campaspe Plains
10.3.28a	851	36	4.3	25	Alice Tableland, Cape Campaspe Plains
10.4.5	85	0	0.0	9	Alice Tableland, Cape Campaspe Plains
10.5.1a	134	3	2.5	2	Alice Tableland, Cape Campaspe Plains
10.5.5a	3030	501	16.5	36	Cape-Campaspe Plains
10.7.11a	189	33	17.2	1	Alice Tableland, Cape Campaspe Plains
11.3.12	214	207	97.0	169	Townsville Plains
11.3.25b	168	127	75.4	90	Townsville Plains
11.3.27	8	6	78.2	3	Townsville Plains
11.3.30	349	268	76.9	534	Townsville Plains
11.3.31	82	73	89.1	14	Townsville Plains
11.3.35	586	491	83.8	527	Townsville Plains
11.3.35a	195	186	95.4	4	Townsville Plains
11.11.9	203	187	92.0	0	Cape River Hills
11.11.15	222	187	84.1	0	Beucazon Hills
11.12.1	365	177	48.6	0	Townsville Plains, Bogie River Hills, Beucazon Hills, Belyando Downs
11.12.9	140	136	96.8	8	Townsville Plains
TOTAL REs	6,821	2,618	38.4	1,423	
HABITAT MODEL	15,563	6,703	43.1	2,233	

**Fig 3:**
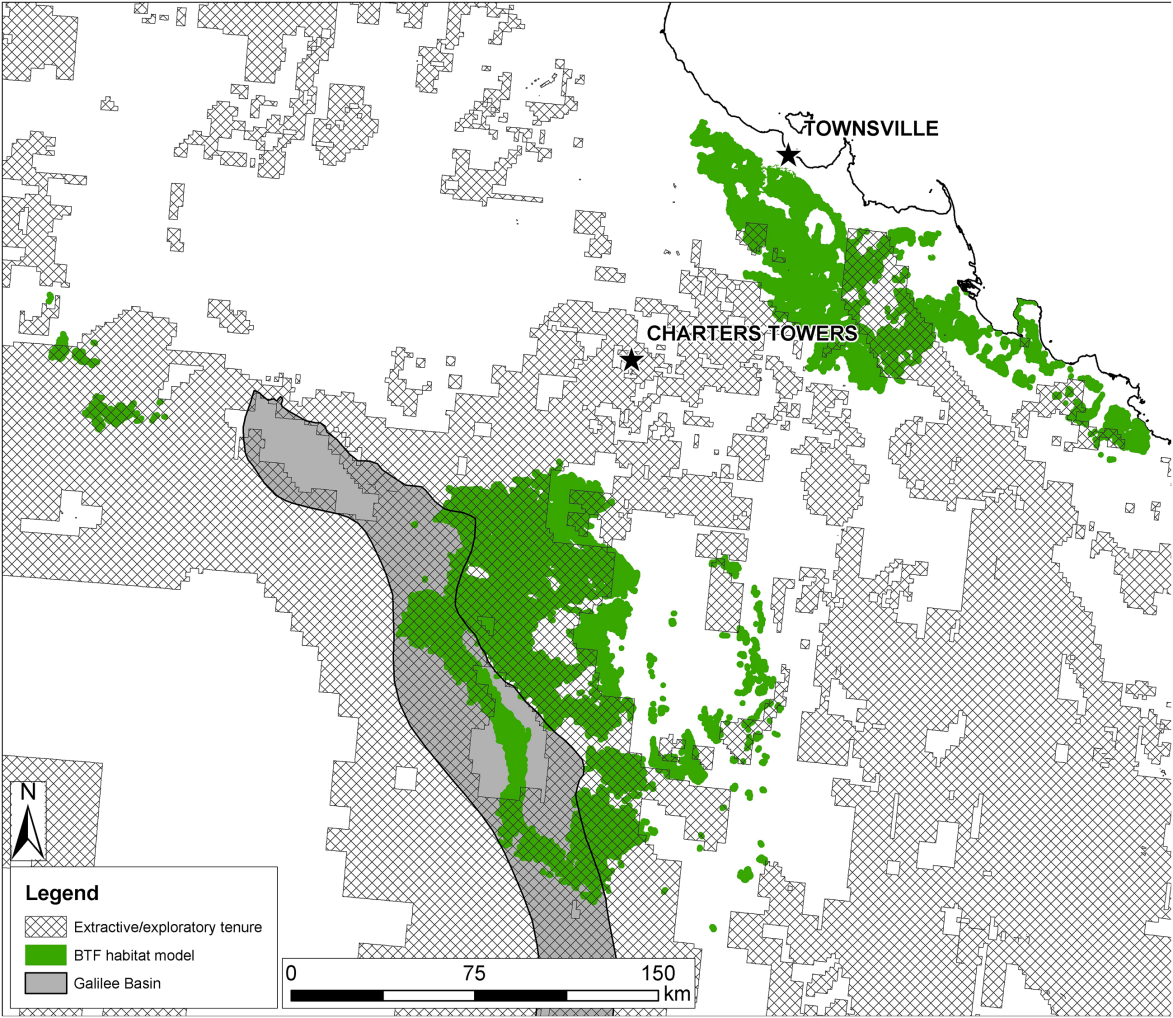
Modelled BTF habitat and extant extractive/exploratory tenures. The BTF habitat model is the favourable BTF REs which are within the area of ≥0.5 bioclimatic threshold climate envelope, buffered to 1118 m. Fig 3 has the same extent and scale as Fig 2. Used under a CC BY license, with permission from Eric Vanderduys, original copyright 2015.

Of the habitat model, 43.1% (6,703 km^2^) remains outside the areas of granted exploration leases or extractive tenure (Fig 3). Protected areas (National Parks) cover 1.5% (235 km^2^) of the habitat model and nine (0.4%) out of 2312 post-2000 BTF records, and each of these records is from the Townsville Plains IBRA subregion. Of the suitable REs within the habitat model, 38.4% (2,618 km^2^) remains outside granted extractive or exploratory industry interests (Table 2). Of EPCs in the Galilee Basin that have been converted to MDLs or MLs, and thus have a higher probability of going ahead as mines, and that have also submitted development plans with infrastructure and mine footprints, 42.5% of the combined original exploration area is likely to be impacted if the mines go ahead as planned (Table 3).

**Table 2:** Total areas for mining tenures and protected areas (National Parks) within the habitat model. Areas are incongruent with figures presented in Table 1 because some areas have more than one mining tenure over them and because of rounding. Habitat model extents as well as actual favourable RE (from Table 1) extents within NPs are also given. Tenures: Exploration Permits for Coal (EPC), Exploration Permits for Geothermal (EPG), Exploration Permits for Mineral (EPM), Exploration Permits for Petroleum (EPP), Mining Claim (MC), Mineral Development Licence (MDL), Mining Lease (ML), Petroleum Lease (PL), Petroleum Survey Licence (PSL).

	Habitat Model
Tenure	Area (km^2^)
EPC	6,756
EPM	1,044
EPP	5,194
MC	0
MDL	11
ML	298
EPG	463
National Park REs	36
National Park (buffered)	235
Total habitat model	15,563

**Table 3:** Total areas for EPCs and MDLs from the southern and central Galilee Basin. Alpha and Kevin's Corner are grouped together because they shared portions of EPC1210. All areas were calculated from [38] and documents referenced in the Source column using ArcGIS 10.1 geo-rectified, low resolution imagery from source documents and creating polygons around affected areas. All areas are given in hectares (Ha). Area affected is the measured impact area within the relevant lease or tenement, broken down into: OC (open cut mine); UG (underground mine); “Other” refers primarily to infrastructure such as buildings and accommodation, waste dumps, sediment dams and airports, but for one project may include an underground mine section (compare Figs 2 and 3 in [86]). EPC = Exploration Permits for Coal, MDL = Mineral Development Licence

Proponent	Lease/Tenement	Area (Ha)	Area affected (Ha)	OC	UG	Other	% affected	Source
Adani	EPC1080 (east portion only)	18,714	32,112^ǂ^	17,424	9,507	5,181	71.8	[81]
	EPC1690	26,016						
Alpha/Kevin's Corner	EPC1210	36,818	50,649	12,969	18,238	19,441	49.7	[39, 82, 87]
	MDL285	33,682						
	MDL333	31,480						
China First	EPC1040	75,674	53,881	71,68	27,630	19,084	38.6	[86]
	EPC1079	63,863						
China Stone	EPC987 (south portion only)	20,066	16,787	35,89	74,04	5,795	83.7	[40]
South Galilee	EPC1049	89,523	14,823	33,47	61,71	5,305	16.6	[41, 83]
TOTAL		395,836	168,252	44,497	68,951	54,805	42.5	

^ǂ^Does not include approximately 2,929 ha industrial area, airport and accommodation village that lie outside extents of EPC1080 and 1690.

For post 2000 BTF records that are beyond the Townsville Plains (and thus likely to be more impacted by mining tenures), 125 of 140 records (89%) are from or within 1118 m of BVG 17b (woodlands to open-woodlands dominated by *Eucalyptus melanophloia* (or *E*. *shirleyi*) on sand plains and footslopes of hills and ranges). The cleared extent of BVG 17b within the three decimal place bioclimatic model threshold is 279 km^2^.

Of the priority areas identified in the GBOS [12], overall 40.0% (18,104 of 45,258 km^2^) falls outside areas with overlying resource tenure: 49.0% of Priority 1; 24.6% of Priority 2; and 39.6% of Priority 3.

## Discussion

The entire habitat of widespread species is rarely threatened by singular events. Rather, small percentage habitat losses, fragmentation and degradation create cumulative impacts resulting in "death by a thousand cuts" [88]. Responsibility for the survival of widespread species may be difficult to define and does not usually fall into the hands of one proponent. The regulatory framework protecting threatened species may be similarly evasive in terms of assigning responsibility. Consequently, decline and extinction of once-widespread species has occurred through multiple factors acting in concert [3].

Our model of BTF habitat shows that 56.9% of the remaining suitable habitat falls within granted, extant resource extraction or exploration tenures (Table 1). Therefore, insufficient BTF habitat exists to secure enough land to offset all the potential extraction or exploration developments. Given that the BTF has lost 80% of its historic range, losing 56.9% of the remaining habitat would be a serious threat to the species' persistence. It is unlikely that all of the extraction or exploration tenure areas will be developed as mines, but for areas with detailed mine plans, 42.5% of the original lease area is planned to be developed. Furthermore, over 80% of the area of favoured REs for BTF along the eastern edge of the Desert Uplands bioregion is under resource extraction or exploration tenures (IBRA subregions: Alice Tableland, Cape Campaspe Plains, Cape River Hills, Undara—Toomba Basalts; Table 1), suggesting that if approximately 42.5% of lease areas are developed, then around 50% of this stronghold is likely to be lost to mining activities. Furthermore, there is a danger that multiple exploratory activities, separate from current planned mines would result in fragmentation, habitat loss and degradation without requiring offsetting, because impacts may be perceived to be insignificant and thus not trigger further investigation.

The Galilee Basin Offset Strategy [12] provides guidance for biodiversity offset planning for the northern Brigalow Belt and Desert Uplands bioregions, which encompass most of the BTF's remaining range. Under the strategy, offsets may be established in degraded or cleared areas that can be improved or rehabilitated in order to actually offset biodiversity losses ([12], p. 21). However, most of the eastern part of the Galilee Basin is held under coal exploration tenure by a number of companies [12, 38] and given that approximately 57% of the modelled BTF habitat could be explored and/or developed for mining, it is technically impossible to apply the current offset arrangements and achieve no net loss of BTF. Little of this key region is available for rehabilitation to offset BTF habitat loss: within the Brigalow Belt, Desert Uplands and Einasleigh Uplands, which collectively provided over 99% of post-2000 BTF records, 43.7% of the climatically suitable area is non-remnant (cleared). The total area of non-remnant land is considerably less than the area under extractive or exploratory tenures (Tables 1 & 2), so there is a deficit of land that could be rehabilitated for BTF habitat offsets. Furthermore, cleared areas of formerly favourable habitat such as open woodlands dominated by *Eucalyptus melanophloia* (BVG 17b) are even more limited. One recently approved mine [89] alone will impact approximately 97 km^2^ of BTF habitat [66]. Therefore, if cleared habitat is to be rehabilitated for offsetting purposes to the Federally required [89] extent of approximately 309 km^2^, then around 28% of the cleared BVG 17b, which is the main favourable habitat impacted, would be required as offsets for this mine alone. Within recorded movement distances of BTF (16 km; Rechetelo, unpublished data, 2014) of this mine's boundary there is less than 43 km^2^ of non-remnant BVG 17b, meaning close proximity offsetting is likely to be impossible [9, 15, 16]. Furthermore, neighbouring applications for additional MDLs and MLs totalling at least 1047 km^2^, are in place [38, 90] further limiting the scope for local offsets.

Another important issue relates to the time lag for restoration to occur [91]. Nowhere within the BTF's range has intentional forward planning occurred to mitigate against time lags (e.g., [66]), nor is it a requirement under the Galilee Basin Offset Strategy [12]. Rather, the purchase or management of offsets usually begins after development commences (e.g., [92]). This strategy can only result in a net loss of habitat or environmental values [43, 93] because it is essential for habitat to be continuously available for persistence of the species; offsets must be created before the activity that they seek to offset is undertaken [6, 93]. To use specific examples from one mine, rehabilitation activities listed in ecofund [94] and GHD [66] are likely to take many years to develop into the high quality habitat they are intended to offset. Hollow-bearing trees, for example, which may be used as nest sites for BTF are likely to take much longer than 30 years to develop [17]. Furthermore, to our knowledge, restoration has not been attempted for BTF habitat in any context. In other systems, restoration of highly degraded habitat often leads to a different ecological community than that which previously existed (e.g., [95–97]). In addition, much of the former BTF range is invaded by buffel grass (*Cenchrus ciliaris*), which is widely favoured by graziers and is highly invasive [98–100] and has never been successfully controlled on a large scale. Where clearing has not occurred, impacts such as grazing are more easily mitigated and thus grazing land managed for BTF could potentially be used as offsets [93]. However, it is not possible to assess this potential aspect of offsetting because specific details are omitted in the Environmental Offset Package ([94], pp. 29–43) and also omitted for an adjacent mine proposal where there is potential overlap of mine developments and offsets [40, 101].

In the Galilee Basin, other threatened species, such as the yakka skink (*Egernia rugosa*), and communities such as RE 10.9.3a (a *Eucalyptus cambageana* woodland), are also likely to be impacted by exploratory or extractive industries to an extent that is difficult or impossible to offset. For example, the entire extent of the *Eucalyptus cambageana* woodland community is within areas of extractive or exploration tenure. For many species and communities, the land available for offsets is limited; so offsets may come in the form of research funding. Although useful for understanding the ecology of the species as a basis for improved conservation, research funding offsets have little direct benefit in actually conserving habitat or protecting the population [4].

Approximately 60% of the area designated by the strategic footprint in the GBOS [12] as potential offsets against loss of biodiversity is itself covered by resource extraction or exploration tenures. Priority 3 areas occupy a greater extent than Priority 1 areas (8,075 km^2^ vs 7,867 km^2^) as they must to adequately offset areas that are in better condition [43, 93]. However, the extent of Priority 1 areas under exploratory or extractive tenure is 8,196 km^2^, whereas extent of Priority 3 areas (potentially to be used as offsets) *not* under exploratory or extractive tenure is 8,075 km^2^, slightly less than the area it is supposed to offset. Thus, the areas actually available for offsets is less than the area that would be required for a 1:1 offset ratio.

Because BTF are primarily ground feeders dependent on seeds accessed on relatively open ground [26], they are vulnerable to habitat alteration by invasive species such as grader grass (*Themeda quadrivalvis*) and shrubby stylo (*Stylosanthes scabra*) (Rechetelo, unpublished data, 2014). Fragmentation is likely to make movement across the landscape more difficult and reduce population viability [30, 32]. Our analysis is likely to underestimate the impacts of the mining developments discussed because we do not account for the impacts of fragmentation, which increase the likelihood of incursion by invasive species, and could change fire regimes, leading to overall lower suitability [3, 102, 103]. Fragmentation would result, for example, from the railway corridors that are planned to service extractive industries in the Galilee Basin; these have not been considered in this paper, but would be long (e.g. 189 km x 95 m [104], approx. 495 km [105]) fragmentation barriers. Also not considered to this point in this paper is potential habitat fragmentation as a result of gas drilling, which has an inherently high edge to footprint ratio because of well spacing and access roads [106]. This is being undertaken at the southern edge of the BTF's current range.

Our results show that adequate provision of offsets to provide protection for BTF is likely to be a difficult proposition in the stronghold area of the eastern Desert Uplands. Protection of remnant high value habitat, while important, should not be considered as offsetting as this will result in a net loss of suitable habitat [43], and protection of offsets if they are restored from cleared or degraded land is likely to be problematic for a number of reasons. First, conditions on approvals (e.g., [107]) require offset areas to be 'legally secured' for at least the duration of the impact [9, 11, 13]. There may be a recommendation of 'in perpetuity' protection [12], but the security of offsets is questionable because they may be revoked [108], Nature Refuges may be developed for mining [86], and even for National Parks, there is currently a designated financial offset ratio (10:1) that may, potentially, be proponent-driven [14] and thus potentially not available for public scrutiny [101]. This policy framework undermines the prospects for secure offset protection for BTF. Second, the ecological requirements of BTF are poorly understood. Although preferred habitats are generally known (see model), there is no established means of rehabilitating heavily degraded or cleared land as BTF habitat. This further undermines the prospects for confidently using offsets as a protection mechanism. Third, timeframes given in offset documents such as 'for the duration of the impact', or 'until 2073' [92], are likely to be insufficient as a long-term protection mechanism and provide little guarantee of offset success [109].

Other land use factors threaten the persistence of the BTF, particularly in other stronghold areas such as the Townsville Plains, where only 34% of the sub-region is planned for resource extraction. The BTF population in this area occurs on the fringe of Australia's largest tropical city and is under threat from ongoing urban expansion, weed invasion, habitat fragmentation and possibly invasive animals. The human population of the Townsville Local Government Area is forecast to expand 122–134% over 2011 levels by 2021, while the broader Townsville region is forecast to expand by 118–128% over the same time frame [110, 111]. There is no reliable BTF population estimate and the large number of records for this region (Fig 1, Table 1) does not equate to a large, stable or secure population but rather proximity to an urban centre with many bird observers, including annual water hole counts since 2004 (BTFRT, unpublished data).

While the plight of the BTF is being considered under federal threatened species legislation, we show here that current mitigation strategies are unlikely to be sufficient to prevent further severe decline. To make a genuine effort to avoid net loss of a species facing habitat loss, stricter protocols such as those proposed by Bos et al. [112] need to be in place. Primarily in the context of groundwater, the Independent Expert Scientific Committee on Coal Seam Gas and Large Coal Mining Development [113] considered that, given the scale of proposed developments within the Galilee Basin, information on cumulative impacts should be commensurate with the scale of all proposed developments. The same holistic approach should be taken when considering development approvals and conditions so that overall risks to a species can be fully evaluated. The area available for offsets is likely to be diminished when many developments occur in one region, and likely to be uncoordinated in the absence of a statutory overarching strategic plan.

Our approach has looked broadly at scope for establishing offsets for BTF in central QLD in the face of planned and prospective broad scale landscape change. Our findings show that there is insufficient existing habitat for BTF that could be used to offset the mining developments planned within its range. Furthermore, insufficient land exists that could be restored to increase the area of habitat. However, even if sufficient land did exist it is unlikely that habitat of sufficient quality could be created within the timeframe required.
